# Vascular Immunotargeting to Endothelial Determinant ICAM-1 Enables Optimal Partnering of Recombinant scFv-Thrombomodulin Fusion with Endogenous Cofactor

**DOI:** 10.1371/journal.pone.0080110

**Published:** 2013-11-14

**Authors:** Colin F. Greineder, Ann-Marie Chacko, Sergei Zaytsev, Blaine J. Zern, Ronald Carnemolla, Elizabeth D. Hood, Jingyan Han, Bi-Sen Ding, Charles T. Esmon, Vladimir R. Muzykantov

**Affiliations:** 1 Department of Pharmacology, Perelman School of Medicine, University of Pennsylvania, Philadelphia, Pennsylvania, United States of America; 2 Center for Targeted Therapeutics and Translational Nanomedicine, Institute for Translational Medicine and Therapeutics, Perelman School of Medicine, University of Pennsylvania, Philadelphia, Pennsylvania, United States of America; 3 Department of Radiology, Perelman School of Medicine, University of Pennsylvania, Philadelphia, Pennsylvania, United States of America; 4 Department of Emergency Medicine, Perelman School of Medicine, University of Pennsylvania, Philadelphia, Pennsylvania, United States of America; 5 Cardiovascular Biology Research Program, Oklahoma Medical Research Foundation, Howard Hughes Medical Institute, Oklahoma City, Oklahoma, United States of America; Emory University School of Medicine, United States of America

## Abstract

The use of targeted therapeutics to replenish pathologically deficient proteins on the luminal endothelial membrane has the potential to revolutionize emergency and cardiovascular medicine. Untargeted recombinant proteins, like activated protein C (APC) and thrombomodulin (TM), have demonstrated beneficial effects in acute vascular disorders, but have failed to have a major impact on clinical care. We recently reported that TM fused with an scFv antibody fragment to platelet endothelial cell adhesion molecule-1 (PECAM-1) exerts therapeutic effects superior to untargeted TM. PECAM-1 is localized to cell-cell junctions, however, whereas the endothelial protein C receptor (EPCR), the key co-factor of TM/APC, is exposed in the apical membrane. Here we tested whether anchoring TM to the intercellular adhesion molecule (ICAM-1) favors scFv/TM collaboration with EPCR. Indeed: i) endothelial targeting scFv/TM to ICAM-1 provides ∼15-fold greater activation of protein C than its PECAM-targeted counterpart; ii) blocking EPCR reduces protein C activation by scFv/TM anchored to endothelial ICAM-1, but not PECAM-1; and iii) anti-ICAM scFv/TM fusion provides more profound anti-inflammatory effects than anti-PECAM scFv/TM in a mouse model of acute lung injury. These findings, obtained using new translational constructs, emphasize the importance of targeting protein therapeutics to the proper surface determinant, in order to optimize their microenvironment and beneficial effects.

## Introduction

A variety of endogenous endothelial proteins project into the vascular lumen and mediate critical homeostatic pathways, helping to maintain blood fluidity, control vascular tone and permeability, and regulate the innate immune response[1]. Several decades of research have demonstrated that loss or functional deficit of these proteins underlies the pathogenesis of a variety of human illnesses[2]–[4]. Molecular therapies capable of replenishing these proteins have the potential to achieve long-sought improvements in the morbidity and mortality of conditions like sepsis and acute lung injury. Two distinct approaches – gene therapy and infusion of recombinant proteins – have been pursued as means to achieve this goal. While the former may be the best suited for long-term correction of chronic deficiencies, temporal considerations make recombinant protein therapeutics the most useful in treating acute vascular disorders.

Results of numerous preclinical and clinical studies support the therapeutic potential of these strategies and provide incentives and directions for further refinements. Since neither transfected gene products nor infused therapeutics typically result in accumulation of proteins at the sites where they exert the optimal effect, achieving proper localization to selected cell types and/or sub-cellular compartments is a key objective for both approaches[5].

Thrombomodulin (TM, CD141) is an endothelial transmembrane glycoprotein, which plays a critical role in regulating inflammation and thrombosis at the vascular margin. TM binds thrombin and blocks its pro-thrombotic and pro-coagulant activities towards fibrinogen, Factor V and protease-activated receptors in platelets and endothelium. Instead, the TM/thrombin complex preferentially cleaves plasma protein C and generates APC, a serine protease with multifaceted anti-thrombotic and anti-inflammatory activities[6].

In this way, TM plays a key role in maintaining vascular homeostasis, matching the pro-coagulant and pro-inflammatory actions of thrombin with the anti-coagulant and anti-inflammatory actions of APC[7]–[9]. This balance is disrupted in a variety of disease states, in which TM is suppressed. Inflammatory mediators, oxidants, and leukocyte proteases have all been implicated in this pathologic process, either through internalization, cleavage, transcriptional regulation, or inactivation of TM on the endothelial surface[10]–[13]. Endothelial TM loss has been demonstrated in human patients suffering from sepsis, atherosclerosis, cardiopulmonary bypass, ischemia/reperfusion injury, and cardiac arrest[14]–[18].

Gene therapy studies in animals have demonstrated beneficial effects of augmenting endothelial expression of TM[19]–[21]. In the acute or emergent setting, where gene therapy is not feasible, infusions of recombinant human APC or soluble TM (sTM) have been tested clinically in patients with severe sepsis, acute lung injury, and disseminated intravascular coagulation[22]–[24]. The initial excitement generated by these untargeted therapeutics, however, has been tempered by their lack of spatiotemporal control, narrow therapeutic window, and limited efficacy[25].

It seems logical to try to anchor TM to its natural site, i.e., the luminal surface of endothelial cells. This has been achieved using vascular immunotargeting directed to a well characterized, safe, and non-internalized endothelial determinant, the platelet endothelial cell adhesion molecule-1 (PECAM-1, CD31). TM fused with an scFv fragment of anti-PECAM binds to endothelium, exerts therapeutic effects, and has a benefit/risk ratio superior to untargeted TM in two separate animal models of acute lung injury[26].

The justification for targeting recombinant TM to endothelial cells is not simply one of pharmacokinetics. Endothelial cells express a key co-factor of the TM/APC system, namely, the Endothelial Protein C Receptor (EPCR, CD201). EPCR markedly enhances APC production by the TM/thrombin complex and mediates endothelial protective and barrier enhancing effects of APC[27], [28]. Endogenous TM and EPCR are believed to be concentrated in the lipid rafts in the endothelial apical plasmalemma[29], [30], whereas PECAM-1 is localized at cell-cell junctions, raising questions about the ability of PECAM-anchored TM to effectively partner with endogenous EPCR[31], [32]. In contrast, the intercellular adhesion molecule-1 (ICAM-1, CD54) is exposed in the apical endothelial plasmalemma, in close proximity to EPCR[33].

Given the critical role that EPCR plays in the endothelial protective effects of the TM/APC pathway, effective partnering with this endogenous co-factor may favor endothelial-targeted TM therapeutics. In this study, we find that recombinant TM anchored to endothelial PECAM-1 does not partner with EPCR, unlike endogenous TM. We describe a new fusion protein, which targets TM to ICAM-1, and show that this endothelial determinant, unlike PECAM-1, allows partnering of recombinant TM with endogenous EPCR. We extend these *in vitro* observations to a model of acute pulmonary inflammation and find that, at an equivalent dose, anti-ICAM scFv/TM provides more robust protection than anti-PECAM scFv/TM. This work reveals the importance of rational selection of cellular targets for biological therapeutics, capitalizing on nanometer-scale membrane topology to allow optimal interaction with endogenous partners.

## Materials and Methods

### Ethics Statement

Animal studies were carried out in accordance with the Guide for the Care and Use of Laboratory Animals as adopted by the NIH, under protocols (803320 and 804349) approved by University of Pennsylvania IACUC.

### Cell lines

YN1 hybridoma and MS1 cells were purchased from ATCC (Manassas, VA). YN1 cells were cultured in RPMI 1640 supplemented with 10% (v/v) fetal bovine serum (FBS). MS1 cells were maintained in DMEM with 10% FBS and 1X antibiotic-antimycotic (Life technologies, Grand Island, NY).

### Antibodies and other reagents

Purified anti-PECAM (390) and anti-ICAM (YN1/1.7.4) antibodies were obtained from BioLegend (San Diego, CA). Anti-TM polyclonal antibody (AF3894) and anti-EPCR polyclonal antibody (AF2749) were purchased from R&D systems (Minneapolis, MN). Anti-EPCR blocking antibody, mAb1560, was supplied by the Esmon laboratory. HRP-conjugated Anti-FLAG (M2-HRP) antibody was obtained from Sigma Aldrich (St Louis, MO). Alexa Flour-labeled secondary antibodies were purchased from Life Technologies (Grand Island, NY). PPACK-inactivated thrombin was a generous gift of Sriram Krishnaswamy. Bovine thrombin, LPS (serotype B4), and mouse TNF were purchased from Sigma. Human protein C zymogen was obtained from Haematologic Technologies (Essex Junction, VT). APC substrate S-2366 was purchased from Diapharma (West Chester, OH).

### Endothelial cell immunofluorescence staining

MS1 cell monolayers were grown in 8 well μ-slides (Ibidi, Verona, WI) and fixed for 20 minutes at room temperature (RT) with Histochoice (Amresco, Solon, OH). In some cases, cells were treated with 10 ng/mL mouse TNF for 8 hours prior to fixation. After three washes, cells were blocked with 3%(w/v) BSA in HBSS for 1 hour at RT. Cells were stained with either anti-PECAM (390, 15 ug/mL) or anti-ICAM (YN1, 1 ug/mL) monoclonal antibodies, in addition to polyclonal goat anti-mouse EPCR (0.5 ug/mL) for 2 hours at RT. Cells were washed three times with 0.1% Tween in HBSS and then stained with Alexa Fluor 594 anti-rat (1∶200) and Alexa Fluor 488 anti-goat (1∶1000). After 1 hour incubation, cells were washed four times with 0.1% Tween in HBSS and once in PBS. ProLong Gold Antifade reagent with DAPI (Life technologies, Grand Island, NY) and a coverslip were applied and cells were allowed to dry overnight prior to immunofluorescence imaging.

### Cloning of anti-ICAM V_L_ and V_H_ cDNAs

Total cellular RNA was isolated from YN1 hybridoma cells using the RNeasy kit (Qiagen, Valencia, CA). Combined reverse transcription and PCR was performed using SuperScript One Step RT-PCR kit (Life Technologies, Grand Island, NY). A single full length V_H_ cDNA was produced using degenerate 5′ framework region 1 (FR1) primers and a 3′ constant region primer[34]. This approach was not possible for the light chain, as PCR utilizing FR1 primers produced only the previously reported Y3-Ag 1.2.3 myeloma V_L_ sequence[35]. An 8 amino acid peptide unique to the ICAM-specific V_L_ was identified using mass spectrometry, and using degenerate primers corresponding to this peptide, a full length V_L_ cDNA was cloned (Figure S1).

### Assembly and expression of anti-ICAM scFv and anti-ICAM scFv/TM constructs

Completed anti-ICAM V_L_ and V_H_ cDNAs were assembled into constructs encoding anti-ICAM scFv and the anti-ICAM scFv/TM fusion protein. In each case, V_H_ and V_L_ sequences were separated by a (GGGGS)_3_ linker, and a triple FLAG tag was appended to the 3′ end (C terminus) for purposes of purification and detection. The anti-ICAM/TM construct was designed to be identical to that of anti-PECAM/TM, with the anti-ICAM scFv on the 5′ end (N terminus), separated from the extracellular domain of TM (amino acids Leu17-Ser517) by an (SSSSG)_2_AAA linker. Both proteins were expressed in S2 cells and purified using a C-terminal triple FLAG tag. Purity was assessed using SDS-PAGE.

### Generation of REN-derived Stable Cell Lines

#### REN-mICAM cells

A full-length cDNA for mouse ICAM-1 was purchased from Thermo Scientific (Rockford, IL). The clone was sequenced and found to contain the entire coding sequence of mouse ICAM-1 and a portion of the 5′ and 3′ UTRs (nt 46-2440) between EcoRI and XbaI restriction enzyme sites. The clone was excised and ligated into the pcDNA3 mammalian expression vector, and transfected into REN cells using Lipofectamine 2000 (Life Technologies, Grand Island, NY). Stably expressing cells were selected in media containing 200 ug/mL of Geneticin (Life Technologies, Grand Island, NY).

#### REN-mPECAM-mEPCR and REN-mICAM-mEPCR cells

A vector containing the entire coding sequence of mouse EPCR and a portion of the 5′ and 3′ UTRs (nt 171–1413) was obtained from the Esmon laboratory[36]. The EPCR cDNA was excised using XbaI and EcoRI and ligated into the pcDNA3.1/Zeo(-) vector (Life Technologies, Grand Island, NY). Since REN-PECAM and REN-ICAM cells already stably express the Geneticin resistance gene, this expression vector (which confers resistance to the antibiotic Zeocin) was utilized. Each cell type was transfected with Lipofectamine 2000 and selected in media with 250 ug/mL of Zeocin.

### Live Cell ELISA Assays

Enzyme-linked Immunosorbent Assays (ELISAs) were performed on live cells as previously described[37], although in the experiments reported here, cell monolayers were incubated with increasing concentrations of scFv or scFv/TM fusion protein rather than whole antibodies. Since all fusion proteins carry a C-terminal triple FLAG tag, anti-FLAG (M2)-peroxidase (HRP) conjugate was used as a detection antibody. In experiments involving MS1 endothelial cells, specific binding of anti-PECAM/TM or anti-ICAM/TM fusion proteins was assessed by co-incubation with 10-fold excess of their parental antibodies (390 and YN1/1.7.4, respectively). ELISA binding data was analyzed and binding parameters (EC_50_) were determined using PRISM 6.0 software (GraphPad, San Diego, CA)[37].

### Radioimmunoassays (RIAs) using 125I-labeled Antibodies

Anti-PECAM (390) and anti-ICAM (YN1/1.7.4) monoclonal Abs were directly radioiodinated with [^125^I]NaI (Perkin Elmer, Waltham, MA) and purified using Zeba desalting spin columns (ThermoScientific). In all cases, radiolabeling efficiency was >75% and free iodine was <5%, post-purification. RIAs were performed and binding parameters (K_d_, B_max_) determined as previously reported[37].

### Protein C Activation Assays

Generation of Activated Protein C (APC) by scFv/TM fusion was assayed as previously described[37]. In soluble APC generation experiments, fusion proteins were mixed with 0.5 nM thrombin and 1μM protein C in a micro-Eppendorf tube, whereas cell-bound assays were conducted by incubating monolayers with scFv/TM fusion protein and washing x 3 with media prior to the addition of 1 nM thrombin and 100 nM protein C. In all cases, protein C activation occurred @ 37C in assay buffer (20 mM Tris, 100 mM NaCl, 1 mM CaCl2, 0.1% (w/v) BSA, pH 7.5) and the reaction was stopped by addition of an excess of hirudin. In experiments involving MS1 cells, the monolayer was first treated with anti-TM antibody to block endogenous TM and then washed x 3 prior to incubation with scFv/TM fusion protein. The amount of APC generated by cell-bound fusions in these experiments was normalized to the number of binding sites per cell, as determined in MS1 RIAs (approximately 240,000/cell for PECAM-1 and 12,000/cell for ICAM-1). In experiments involving EPCR blockade, cells were incubated with 300 nM of anti-EPCR antibody (Ab1560) for 15 minutes prior to the addition of protein C and thrombin. This antibody has been well characterized and is known to inhibit approximately 70% of protein C binding, eliminating to substantial extent the ability of EPCR to accelerate the activation of protein C by the thrombin-TM complex [38].

### Intratracheal (IT) LPS Model

C57BL/6 male mice weighing 20-30 g were anaesthetized and placed in a supine position. Acute lung injury was induced via IT injection of 2 mg/kg of endotoxin in a volume of 100 uL of PBS. Endotoxin injection was followed immediately by injection of 150 uL of air, to ensure even distribution of LPS throughout all distal airspaces. Anti-PECAM scFv/TM, anti-ICAM scFv/TM, or PBS vehicle were injected intravenously 30 minutes prior to LPS administration. In relevant experiments, ^125^I-labeled albumin was also injected intravenously 5 minutes prior to LPS administration. 6 hours after induction of lung injury, blood was withdrawn from the inferior vena cava and animals were euthanized. In experiments involving tracing of ^125^I-labeled albumin, a catheter was placed in the pulmonary artery and the pulmonary circulation was gently flushed with PBS prior to the harvesting of organs. Bronchoalveolar lavage was performed via a 19-gauge stainless steel catheter (Harvard Apparatus, Holliston, MA) placed in the trachea and secured via a 5-0 silk suture. Each animal was lavaged twice with 0.8cc of ice-cold PBS. The lavages were pooled and MIP-2 was quantified using a Quantikine ELISA kit (R&D systems, Minneapolis, MN). For quantification of VCAM-1 and E-selectin mRNA, lungs were homogenized with steel beads (Sigma) and a Tissue Lyser II (Qiagen). Total RNA was isolated with RNeasy kit and cDNA was synthesized using the Transcriptor 1^st^ Strand cDNA Synthesis Kit (Roche Applied Science, Indianapolis, IN). qPCR was performed using the FastStart DNA MasterPLUS kit (SYBR green) and a Lightcycler 1.5 carousel-based system (Roche Applied Science). Validated Quantitect primers for mouse VCAM-1, E-selectin, and actin (housekeeping control) were utilized (Qiagen, Valencia, CA).

### Data analysis and statistics

Results are expressed as mean ± SD unless otherwise noted. Significant differences between means were determined using one-way ANOVA followed by appropriate multiple comparison (Tukey) test. P<0.05 was considered statistically significant.

## Results

### PECAM-1 vs. ICAM-1: Relative proximity to EPCR and number of binding sites on endothelial surface

**Figure 1:**
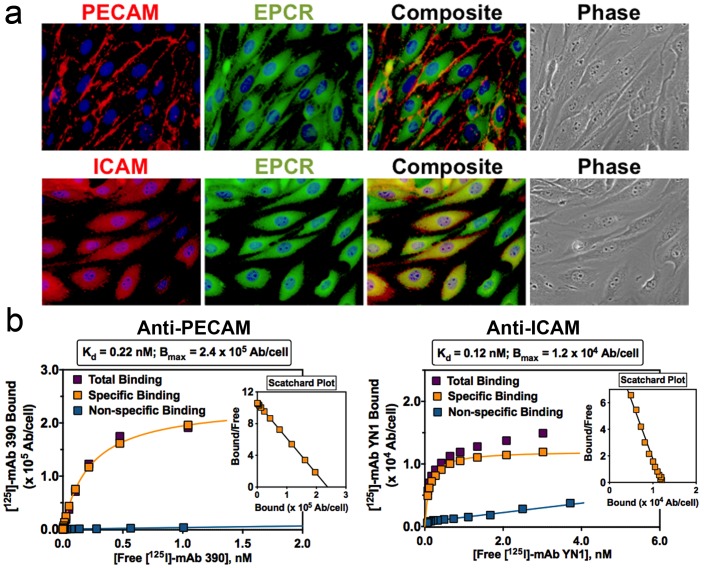
Localization of PECAM-1, ICAM-1, and EPCR on MS1 mouse endothelial cells. (a) Immunofluorescence images demonstrate superior co-localization of ICAM-1 and EPCR, as compared to PECAM-1 and EPCR. (b) Radioimmunoassay data using ^125^I-labeled anti-PECAM and anti-ICAM parental antibodies to determine affinity and number of binding sites per endothelial cell.

In order to assess the relative proximity of EPCR to PECAM-1 and ICAM-1, mouse MS1 endothelial cells were stained for each antigen and imaged using a fluorescence microscope (Figure 1a). In agreement with previous reports, most PECAM-1 staining occurred at cell-cell borders[31], with minimal overlap with EPCR. In contrast, there was gross overlap of staining for ICAM-1 and that for EPCR (Figure 1a).

Next, we studied binding to MS1 cells of ^125^Iodine-labeled PECAM and ICAM antibodies used to produce scFv to fuse with TM. Anti-ICAM and anti-PECAM had similar high affinities (K_d_ of approx. 0.12 and 0.22 nM, respectively), although PECAM-1 provided ∼20-fold more binding sites than ICAM-1 (approximately 240,000/cell vs. 12,000/cell, respectively), reflecting a substantial difference in the level of cell surface expression of these molecules (Figure 1b). According to these data, MS1 have a lower number of anti-PECAM binding sites than human umbilical vein endothelial cells (HUVECs, ∼10^6^ binding sites per cell[39]), which may reflect innate differences between these cell lines. In particular, MS1 cells are smaller than HUVEC, so their number of PECAM copies per cell surface area may be fairly similar.

### Binding of ICAM and PECAM-targeted scFv/TM fusion proteins

**Figure 2:**
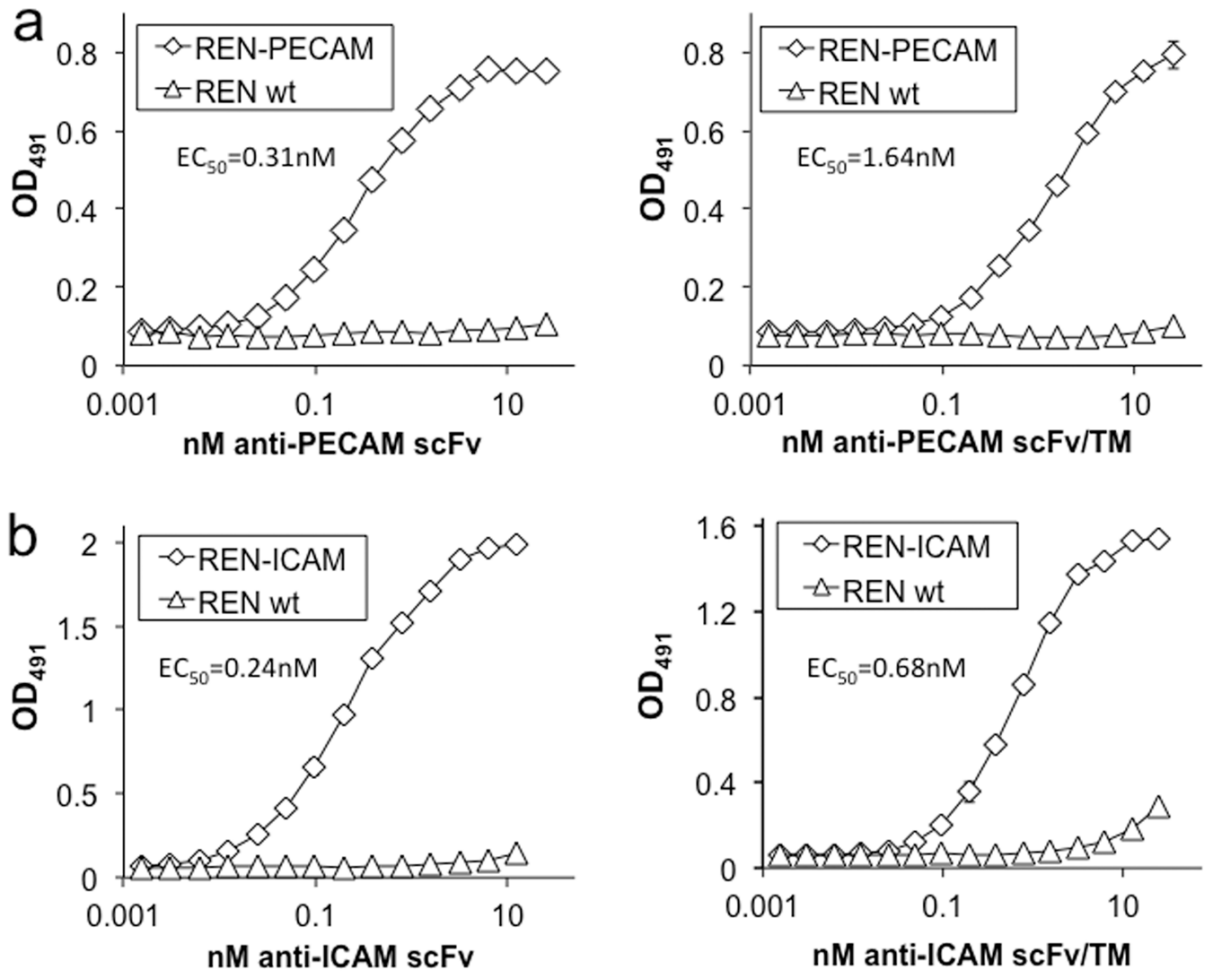
Binding of ICAM and PECAM-targeted scFv and scFv/TM fusion proteins. Cell based ELISAs show binding of (a) anti-PECAM scFv and anti-PECAM scFv/TM fusion protein to PECAM expressing REN cells, and (b) anti-ICAM scFv and anti-ICAM scFv/TM fusion protein to ICAM expressing REN cells. No significant binding is seen to wild type REN cells. Experiments were done in triplicate (each point shown represents three wells), with SD shown. EC50 is shown for each curve.

We next constructed an anti-ICAM-1 antibody fragment (scFv) and fused it to the extracellular domain of TM (Figure S1). In order to compare the binding of the anti-PECAM/TM and anti-ICAM/TM fusion proteins, we utilized REN cells stably expressing either mouse PECAM-1 or ICAM-1. The human mesothelioma cell line REN is a useful model system, with no expression of mouse PECAM, ICAM, TM, or EPCR at baseline (Figure S2). Both anti-PECAM scFv and anti-ICAM scFv, as well as their respective TM fusion proteins, demonstrated sub-nanomolar affinity to REN cells expressing their corresponding target molecule. Little or no non-specific binding was seen to wild type REN cells (Figure 2).

### Functional Activity of PECAM and ICAM-targeted TM fusion proteins in solution and on non-endothelial REN cells

We next assessed the TM activity of the anti-PECAM/TM and anti-ICAM/TM fusion proteins. When tested in a fluid-phase assay, the fusion proteins were nearly identical to soluble TM in their ability to stimulate thrombin-mediated activation of protein C (Figure S3).

**Figure 3:**
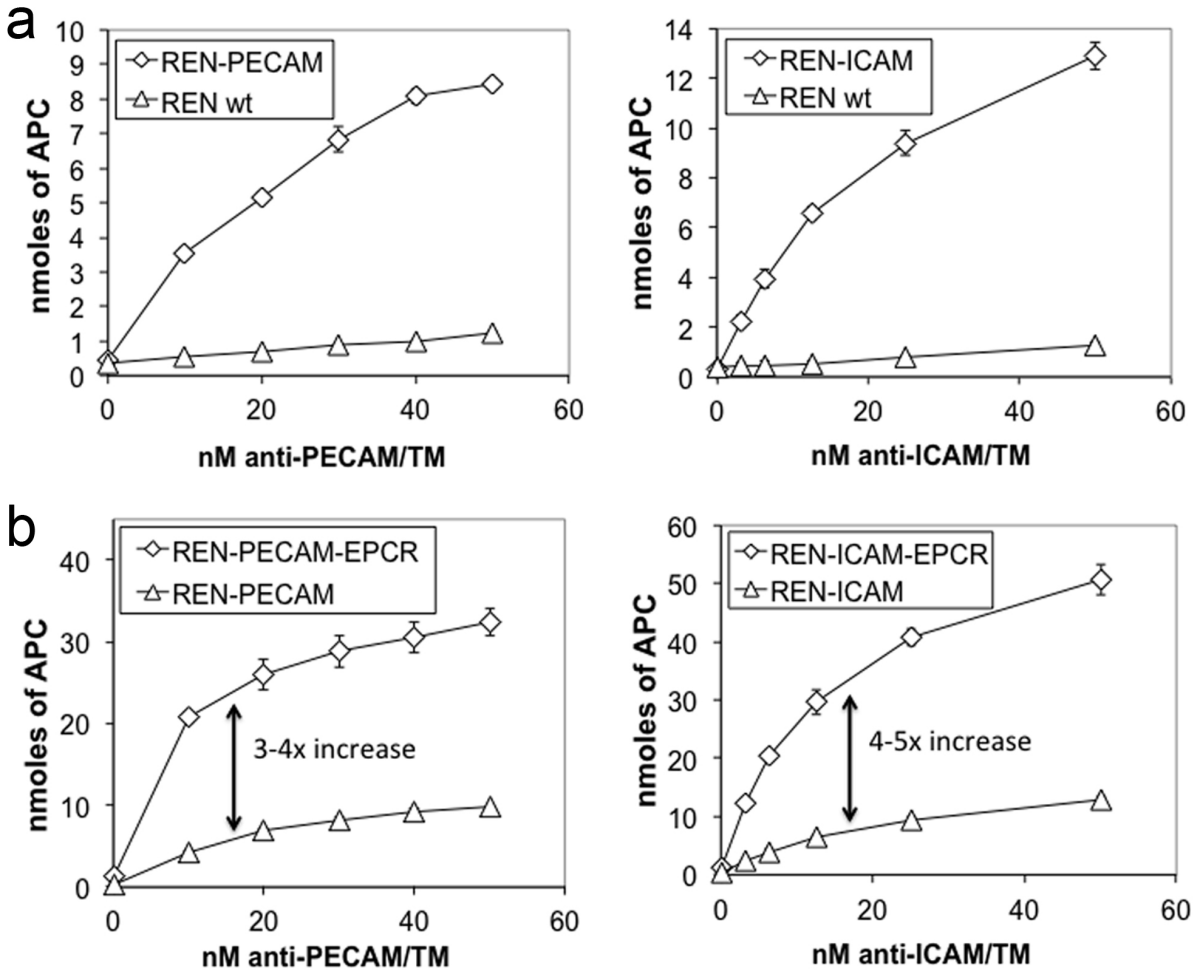
APC generation by TM fusion proteins on non-endothelial REN cells with and without EPCR expression. (a) anti-PECAM scFv/TM and anti-ICAM scFv/TM activate protein C while bound to PECAM and ICAM-expressing cells, respectively. Minimal APC is generated on wild type REN cells, presumably due to lack of binding. (b) A ∼4-fold increase in APC generation is seen when PECAM and ICAM-targeted TM fusion proteins are anchored to cells which stably express mouse EPCR (i.e. REN-PECAM-EPCR and REN-ICAM-EPCR cells), as compared to EPCR-negative counterparts. All experiments were done in triplicate. Data shown are mean ± SD.

In the next series of experiments, we used an assay for measuring the activation of protein C on the surface of cells[37] to test the activity of anti-PECAM/TM and anti-ICAM/TM while bound to REN cells expressing their corresponding target antigen. Each fusion protein demonstrated dose-dependent, thrombin-mediated activation of protein C on cells expressing their target ligand, but not on wild type REN cells (Figure 3a). sTM showed no activity on any cell type, presumably due to lack of binding. Cell-bound anti-ICAM/TM demonstrated greater APC production than anti-PECAM/TM, in accord with a higher number of binding sites on REN-ICAM vs. REN-PECAM cells (8.7×10^5^ vs. 2.6×10^5^ binding sites per cell, respectively, Figure S4).

### EPCR expression potentiates the functional activity of both PECAM and ICAM-targeted fusion proteins on non-endothelial cells

Having determined the baseline rate of protein C activation by each fusion protein on model cells expressing their corresponding anchoring molecule, we next assessed the general ability of cell-bound fusions to partner with EPCR in the membrane. To achieve this, EPCR expression was induced on REN-PECAM and REN-ICAM cells, producing the stable cell lines REN-PECAM-EPCR and REN-ICAM-EPCR (Figure S2). EPCR expression resulted in ∼4-fold enhancement of thrombin-mediated APC generation by both anti-PECAM/TM and anti-ICAM/TM (Figure 3b). In summary, while bound to their corresponding anchors on non-endothelial REN cells, PECAM and ICAM-targeted scFv/TM fusion proteins demonstrated roughly equivalent functional activity and similar capacity to partner with cellular EPCR, at least with respect to thrombin-dependent APC generation.

### Binding and Protein C activation by PECAM and ICAM-targeted fusion proteins on endothelial cells

While transfected REN cells are convenient for studying TM fusion proteins, they clearly represent an artificial system, in which the surface expression and distribution of PECAM, ICAM, and EPCR do not necessarily reflect what is present on endothelial cells. Indeed, RIA using ^125^I-labeled anti-EPCR revealed that the number of binding sites on the REN-PECAM-EPCR and REN-ICAM-EPCR stable cell lines was an order of magnitude higher than on MS1 mouse endothelial cells (Figure S5).

**Figure 4:**
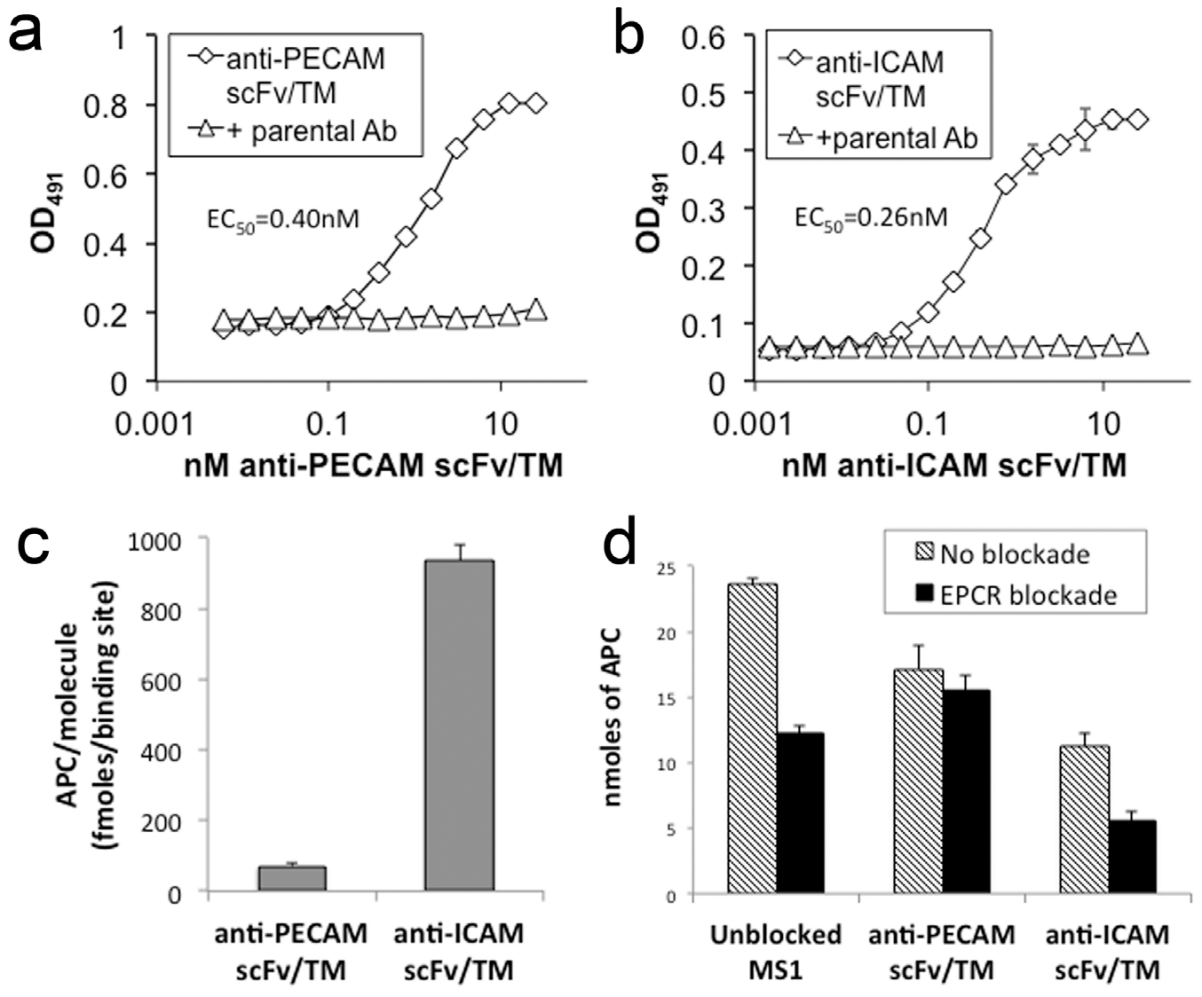
Binding and activity of TM fusion proteins on mouse endothelial cells. (a) Anti-PECAM scFv/TM and (b) anti-ICAM scFv/TM bind to their respective ligands on MS1 cells. Binding is inhibited by excess of parental anti-PECAM-1 and anti-ICAM-1 antibodies. (c) Anti-ICAM scFv/TM demonstrates ∼15-fold greater activity per binding site on MS1 cells, as compared to its PECAM-anchored counterpart. (d) Antibody blockade of EPCR results in a ∼50% decrease in APC generation by endogenous TM and anti-ICAM scFv/TM, but not anti-PECAM scFv/TM. All experiments were done in triplicate. Data shown are mean ± SD.

Accordingly, the binding and functional activity of anti-PECAM/TM and anti-ICAM/TM were next tested on MS1 cells. Each fusion protein demonstrated specific binding to its target ligand, as evidenced by near complete inhibition of binding by a 10-fold excess of parental antibody (Figure 4a,b). Calculated affinity constants were similar to those seen in previous experiments using REN cells transfected with PECAM and ICAM (EC_50_ of 0.4 nM for anti-PECAM/TM and 0.26 nM for anti-ICAM/TM).

Measuring the activity of the fusion proteins on endothelial MS1 cells is substantially more complicated than on REN cells, due to high level of expression of endogenous TM. In order to measure fusion protein-specific APC generation on endothelial cells, we blocked the activation of protein C by endogenous TM. Both PPACK-inactivated thrombin and a polyclonal anti-TM antibody were able to fully inhibit the activity of endogenous TM. Ultimately, we used the antibody as a blocking agent since it had a sustained effect after washing. Sustained blockade of 60-70% of endogenous TM activity, provided by anti-TM antibody, enabled measurement of dose responsive, fusion protein-dependent protein C activation (Figure S6).

We employed these conditions to assess protein C activation by anti-PECAM/TM vs. anti-ICAM/TM anchored to MS1 cells. The amount of APC generated by cell-bound fusions was normalized to the number of binding sites per cell. This analysis revealed ∼15-fold greater APC generation by anti-ICAM/TM vs. anti-PECAM/TM (Figure 4c). To assess the role of EPCR in this marked difference in fusion protein activity, we blocked EPCR interaction with murine Protein C using a monoclonal antibody that thereby inhibits its ability to accelerate APC production by the thrombin-TM complex[38]. Indeed, we found that treatment of MS1 cells with the anti-EPCR antibody resulted in approximately 50% reduction in thrombin-dependent activation of protein C by endogenous TM (Figure 4d). We observed a similar 50% reduction in the activation of protein C by anti-ICAM/TM on MS1 cells, following blockade of endogenous TM. In contrast, there was no significant effect on the activation of protein C by anti-PECAM/TM (Figure 4d).

These results, together with the 15-fold difference in APC generation by the fusion proteins on endothelial cells, indicate that recombinant scFv/TM partners more effectively with endogenous EPCR when anchored to ICAM, as opposed to PECAM.

### Endothelial protective effects of PECAM and ICAM-targeted fusion proteins in a mouse model of acute lung injury

**Figure 5:**
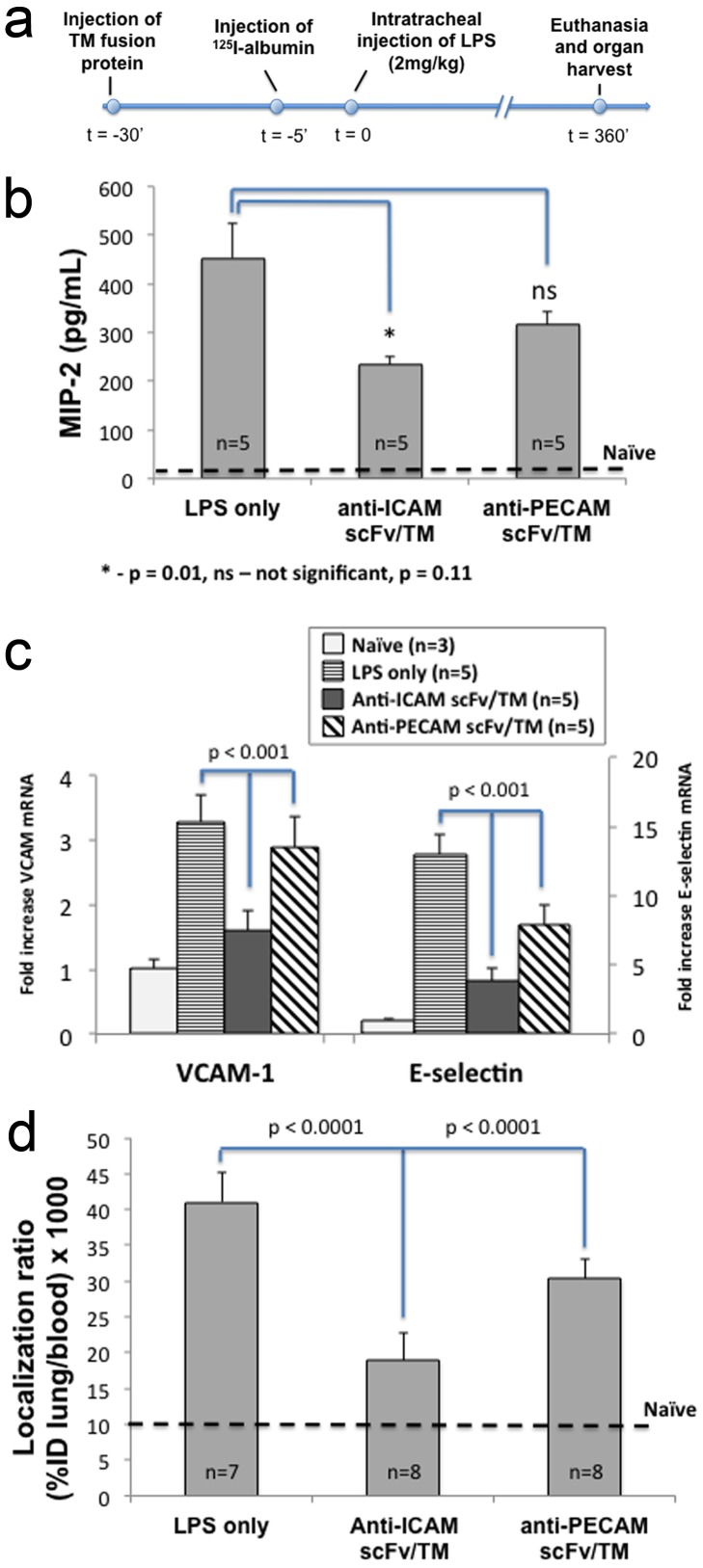
Anti-ICAM scFv/TM provides enhanced endothelial protection in a mouse model of lung inflammation/injury. (a) Timeline of intratracheal LPS lung injury model. In experiments assessing endothelial barrier dysfunction, a tracer amount of ^125^I-labeled albumin was injected 5 minutes prior to LPS administration. (b) Concentration of the chemokine MIP-2 in bronchoalveolar lavage (BAL) fluid. (c) mRNA transcript levels of pro-inflammatory cell adhesion molecules, VCAM-1 and E-selectin, in lung homogenate. (d) Endothelial barrier dysfunction, as measured by leakage of ^125^I-labeled albumin from blood into lung interstitium and/or alveolar space. All data shown are mean ± SD, with number of animals as shown.

Anti-inflammatory effects of anti-PECAM/TM and anti-ICAM/TM were then compared in a model of lung inflammation, in which mice receive an intratracheal injection of endotoxin. The fusion proteins, or PBS vehicle, were injected intravenously 30 minutes prior to LPS challenge (Figure 5a). Relevant indices of lung inflammation and injury were measured, including the level of MIP-2 in bronchoalveolar lavage fluid (Figure 5b), expression of cell adhesion molecules VCAM-1 and E-selectin in lung tissue homogenate (Figure 5c), and extravascular leakage of radiolabeled albumin injected intravenously and detected in the lungs (Figure 5d). While both anti-PECAM/TM and anti-ICAM/TM showed evidence of protection, the ICAM-targeted fusion protein was more effective in all cases.

## Discussion

Thrombomodulin is a multifaceted mediator of vascular homeostasis. For reasons that are not fully understood, humans and other mammals have evolved mechanisms by which the TM/protein C pathway is suppressed in a variety of settings, tipping the balance towards thrombosis, leukocyte adhesion, and vascular leak. While endothelial TM loss may have selective advantage in some situations, such as localized infection or trauma, there is substantial evidence to suggest that it has a role in the pathogenesis of a variety of contemporary human illnesses[14]–[18].

The direct reversal of this process through gene therapy has provided proof-of-principle for replenishing endothelial TM[19]–[21]. In chronic settings such as atherosclerosis and vascular bypass grafting, genetic approaches may be preferable[40], but they are unlikely to have a role in unforeseeable and emergent conditions like sepsis, stroke, and acute lung injury. The landmark discovery that recombinant human APC could protect baboons from a otherwise lethal infusion of E Coli raised expectations that this approach could restore the critical TM/APC pathway in sepsis and other diseases[41]. However, infusion of an activated zymogen lacks the “on demand” nature of the endogenous TM/protein C response, and clinical utility has been limited by a narrow therapeutic window and serious systemic side effects[25]. Likewise, infusions of soluble TM – or various genetic mutants – have shown initial promise in animal models and human clinical trials[24], [42], but do not allow recombinant TM to capitalize on the unique factors of the endothelial luminal microenvironment critical to its function.

Vascular immunotargeting is a modern strategy to direct the delivery and therapeutic effect of drugs via their conjugation to specific affinity ligands of determinants on the luminal surface of endothelial cells. Several promising endothelial targets have been explored, including cell adhesion molecules, caveolar aminopeptidase P, angiotensin converting enzyme, and aminopeptidase N[43]–[48]. This strategy has allowed marked improvement of therapeutic interventions in numerous animal models of human diseases, but further improvements are warranted. Specifically, it has become clear that binding to target cells is necessary, but not always sufficient, for optimal results. In many cases, the cargo needs even more precise addressing at sub-cellular level, such as internalization and accumulation in selected organelles[5], [49].

Protein therapeutics that exert their activity in the vascular lumen, such as thrombomodulin, are a special case in which the cargo must be retained on the surface, instead of entering the cells. Ligands such as PECAM-1 and ICAM-1, endothelial adhesion molecules with limited internalization, represent preferable targets. We have now devised scFv/TM fusions directed to each of these determinants. The results of the present study, for the first time comparing these fusions, indicate that scFv/TM functionality and protective effect benefit from a nanoscale level of sub-cellular localization – not only targeting the surface membrane, but a particular determinant which allows cooperation of the cell-anchored fusion with endogenous endothelial cofactor(s).

**Figure 6:**
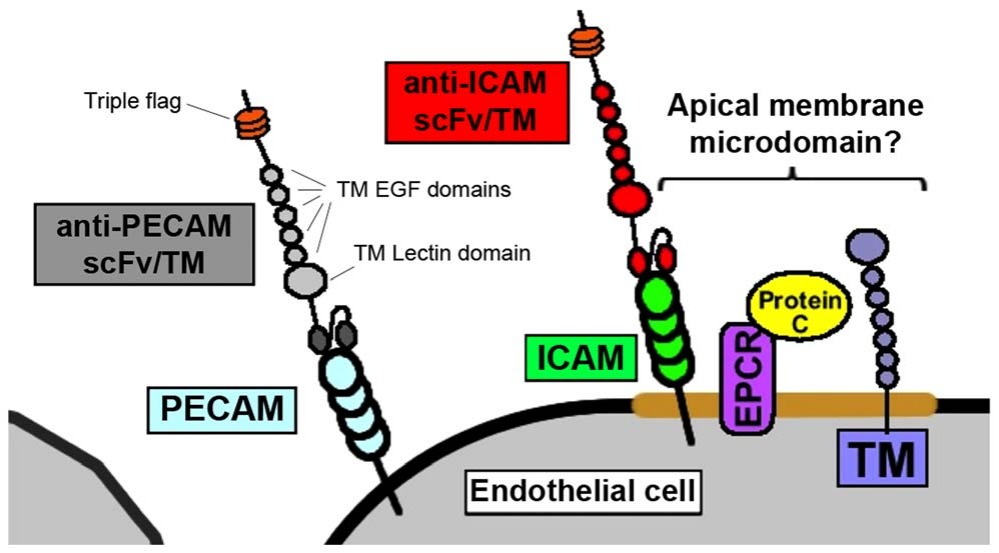
Schematic representation of TM fusion proteins anchored to the endothelial plasmalemma. The proximity of ICAM-targeted TM to endogenous EPCR/Protein C may account for its enhanced activity *in vitro* and *in vivo*.

The most important cofactor, in the case TM and APC, is the endothelial protein C receptor[27]. In addition to accelerating the activation of protein C, EPCR plays an critical role in the endothelial protective signaling of APC and is necessary for the protective effects of APC in diseases of endothelial injury and dysfunction, such as severe sepsis[28], [50]. The current data set supports several conclusions: 1. the ability of scFv/TM fusion protein to interact with endogenous EPCR depends on which surface determinant is targeted (Figure 4), and 2. this variable has significant therapeutic implications, with the ICAM-targeted scFv/TM fusion demonstrating more potent protective effects *in vivo* (Figure 5). Our data, along with prior reports regarding the distribution of ICAM and PECAM on the endothelial membrane[31]–[33], suggest that the proximity of the TM fusion to EPCR may be the critical factor. Figure 6 shows a simplified model of an endothelial cell with the TM fusion proteins bound to their target ligands. Of note, the figure accurately depicts the fusion proteins binding the domains of PECAM-1 and ICAM-1 which lie furthest from the plasma membrane, consistent with the location of their target epitopes[37], [51]. The schematic highlights the proposed difference in proximity to the EPCR/Protein C complex, which may account for our experimental observations.

It is not known if partnering between anti-ICAM/TM and EPCR is limited to the stimulation of protein C activation, or what role EPCR may have in the endothelial protective effects of TM fusion proteins. In theory, one would expect the requirement for molecular proximity to be less stringent in mediating signaling by APC generated by endothelium-anchored fusion protein, which might interact with and signal through EPCR in a paracrine manner. This intriguing issue is worth further investigation. Likewise, a rigorous appraisal of the benefit/risk ratio of the TM fusion proteins is needed in relevant animal models of human diseases, with administration both prior to and after the onset of injury.

It is worth noting that the results of this study align with the general notion that anchoring agents to distinct determinants on the same target cell may produce distinct outcomes, due to the differing functions, location, and trafficking of these surface molecules. For example, our laboratory previously reported that binding of the H_2_O_2_-producing enzyme, glucose oxidase (GOX), to endothelial cells induced varying degrees of vascular damage, depending on whether PECAM or TM was chosen as the surface target[52]. The variation in outcome in those experiments was attributed to the substantially different function of these two surface molecules and the consequences of their blockade by GOX conjugates. In contrast, it is difficult to attribute the current results to any functional difference between ICAM and PECAM, two closely related proteins which both support leukocyte adhesion, pro-inflammatory signaling, and uptake of antibody conjugates via a similar endocytic mechanism[53], [54]. For this reason, we believe that the most logical explanation for our current experimental results is the distinct localization of ICAM and PECAM on the endothelial membrane and their differing capacity to allow interaction of anchored scFv/TM with EPCR.

In summary, we report the creation of a novel ICAM-targeted TM fusion protein and evidence for it’s partnering with endogenous endothelial EPCR in the activation of protein C. Delivery of recombinant TM to the endothelial membrane in a way that mimics its natural distribution and allows interaction with endogenous co-factors represents a new strategy for restoration and/or augmentation of the TM/Protein C pathway and may provide effective treatment of a wide variety of human illnesses.

## Supporting Information

Figure S1 **Cloning and assembly of anti-ICAM scFv and scFv/TM fusion protein.**
(PDF)

Figure S2 **Creation of REN cells stably expressing PECAM and EPCR.**
(PDF)

Figure S3 **APC generation by TM fusion proteins in fluid phase assay.**
(PDF)

Figure S4 **Quantification of ICAM and PECAM binding sites on transfected REN cells.**
(PDF)

Figure S5 **Quantification of EPCR binding sites on transfected REN cells.**
(PDF)

Figure S6 **APC generation on MS1 cells following blockade of endogenous TM.**
(PDF)
