# Supplementary material for: Vascular Immunotargeting to Endothelial Determinant ICAM-1 Enables Optimal Partnering of Recombinant scFv-Thrombomodulin Fusion with Endogenous Cofactor
Source: PLoS One. 2013 Nov 14;8(11):e80110. doi: 10.1371/journal.pone.0080110 (PMC3828233; doi:10.1371/journal.pone.0080110)
Supplement: Figure S1 — Cloning and assembly of anti-ICAM scFv and scFv/TM fusion protein. (PDF) [file pone.0080110.s001.pdf]

Figure S1

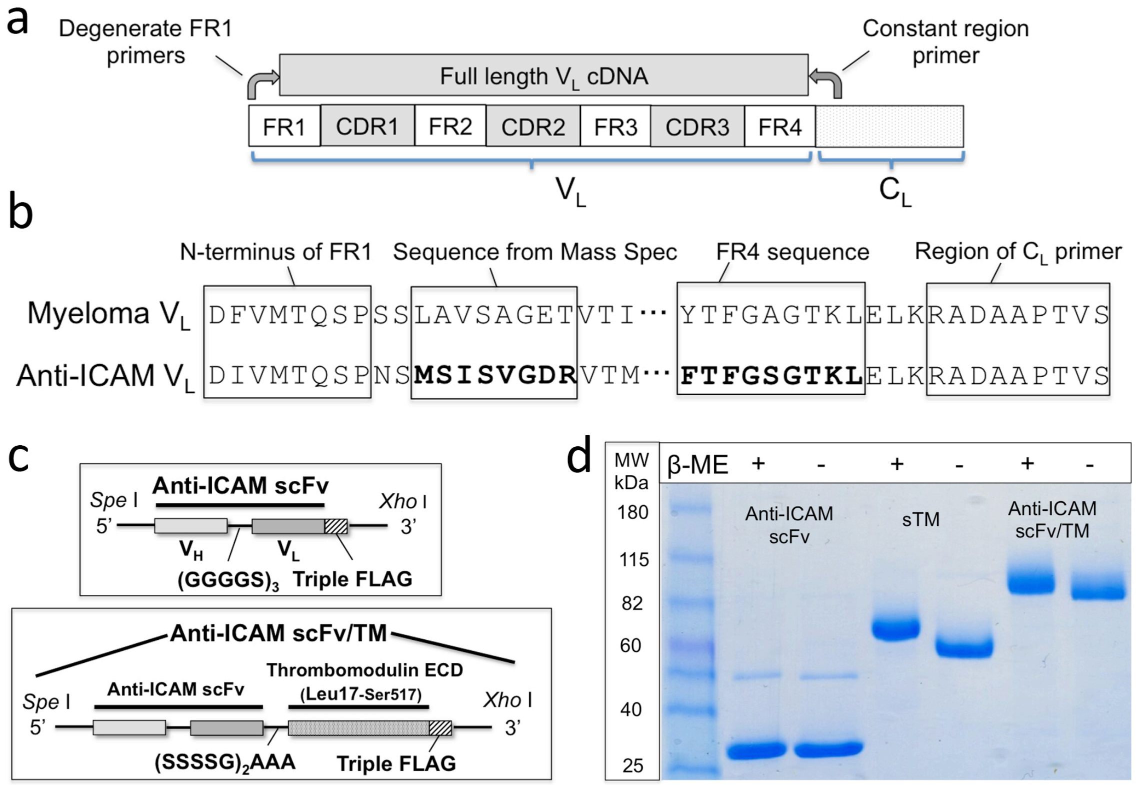

Supplemental Figure 1. Cloning and assembly of anti-ICAM scFv and scFv/TM fusion protein. (a) The typical approach to PCR cloning of variable heavy and light chain regions (V_H and V_L) utilizes degenerate 5' primers corresponding to the beginning of the FR1 region and a 3' primer corresponding to the start of the constant region. (b) In the case of the YN1/1.7.4 anti-ICAM hybridoma, the typical approach amplified only the myeloma V_L, which is nearly identical to the anti-ICAM V_L at the N-terminus of FR1 region. Mass spectrometry was used to identify an 8 amino acid sequence unique to the anti-ICAM V_L. Degenerate primers were synthesized and paired with the 3' constant region primer. A second 3' primer was synthesized (corresponding to the 9 amino acid FR4 sequence shown) and paired with the original set of degenerate 5' primers to identify the residues at the N-terminus of the anti-ICAM V_L. (c) Assembly of V_H and V_L sequences into anti-ICAM scFv and scFv/TM constructs. (d) SDS PAGE gel electrophoresis of anti-ICAM scFv, soluble TM, and anti-ICAM scFv/TM fusion protein under reducing and non-reducing conditions.
