# Supplementary material for: Vascular Immunotargeting to Endothelial Determinant ICAM-1 Enables Optimal Partnering of Recombinant scFv-Thrombomodulin Fusion with Endogenous Cofactor
Source: PLoS One. 2013 Nov 14;8(11):e80110. doi: 10.1371/journal.pone.0080110 (PMC3828233; doi:10.1371/journal.pone.0080110)
Supplement: Figure S2 — Creation of REN cells stably expressing PECAM and EPCR. (PDF) [file pone.0080110.s002.pdf]

Figure S2

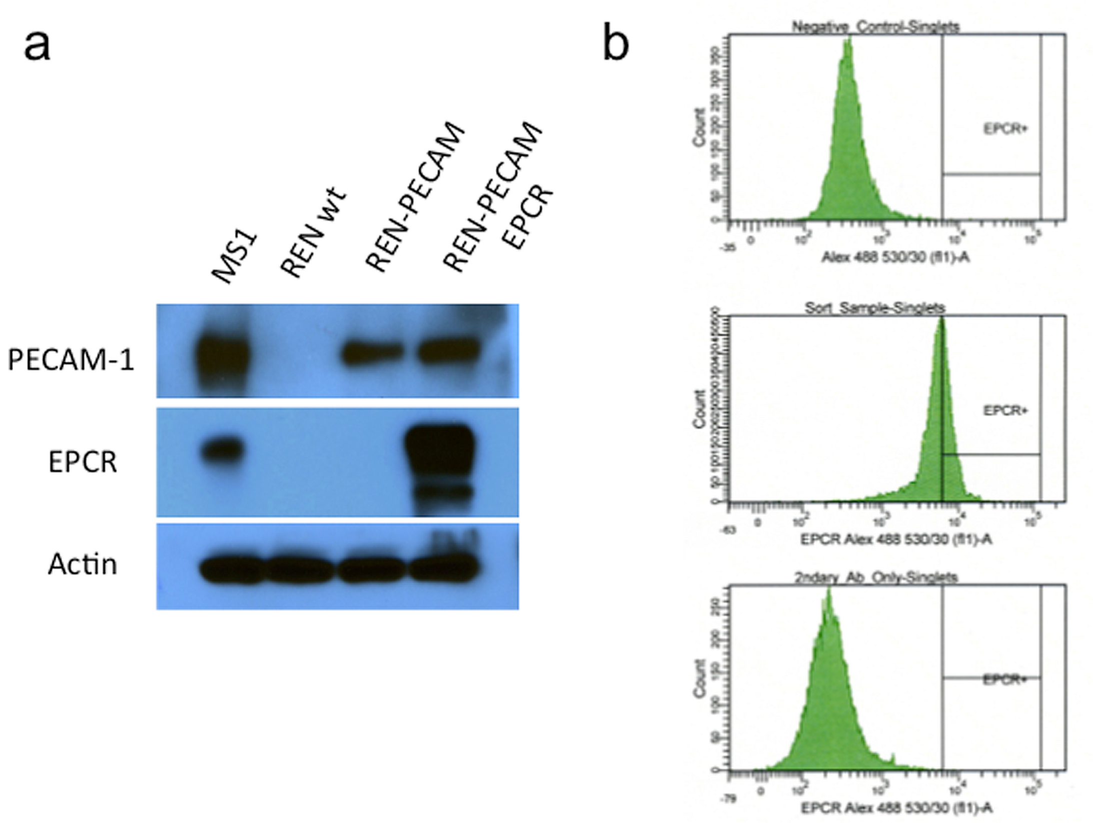

Supplemental Figure 2. Creation of REN cells stably expressing PECAM and EPCR. (a) REN cells are a human mesothelioma cell line with no baseline expression of mouse PECAM-1, ICAM-1, TM or EPCR. REN cells stably expressing mouse PECAM-1 (REN-PECAM) were transfected with a vector containing mouse EPCR and the zeocin resistance gene. REN-PECAM cells, which are normally maintained in medium containing geneticin, were doubly selected with geneticin and zeocin. Expression of was confirmed by western blotting. (b) Flow cytometry was used to confirm surface localization. Middle panel shows histogram of REN-PECAM-EPCR cells stained with anti-EPCR primary and Alexa Fluor 488-secondary antibody. Upper and low panels show negative controls – ie. untransfected REN-PECAM cells and REN-PECAM-EPCR cells stained with secondary antibody only. A similar procedure was followed to obtain REN cells expressing ICAM and EPCR.
