# Supplementary material for: Vascular Immunotargeting to Endothelial Determinant ICAM-1 Enables Optimal Partnering of Recombinant scFv-Thrombomodulin Fusion with Endogenous Cofactor
Source: PLoS One. 2013 Nov 14;8(11):e80110. doi: 10.1371/journal.pone.0080110 (PMC3828233; doi:10.1371/journal.pone.0080110)
Supplement: Figure S3 — APC generation by TM fusion proteins in fluid phase assay. (PDF) [file pone.0080110.s003.pdf]

Figure S4

a

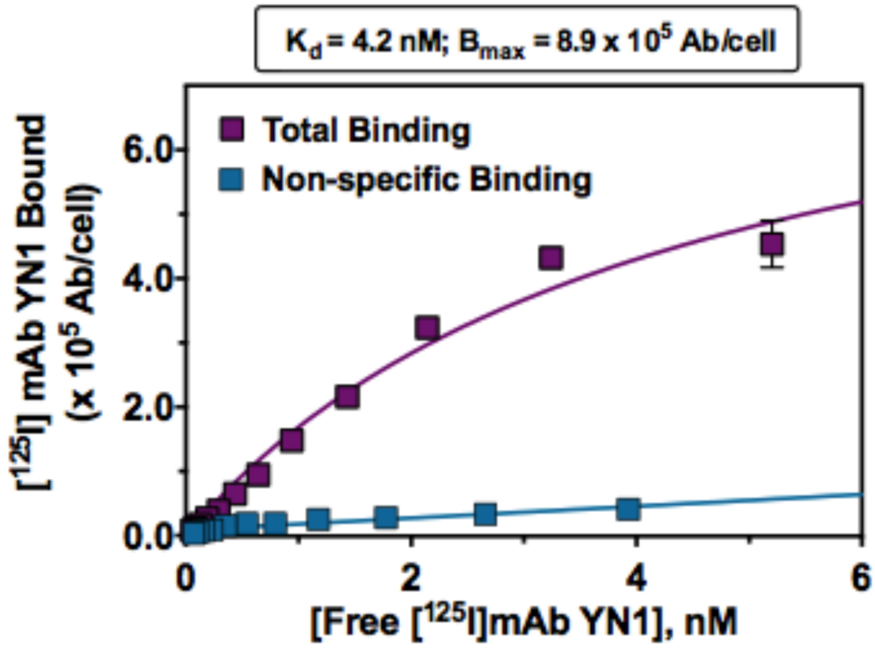

b

Cells	mAb	B_{max} (Ab/cell)
REN-PECAM[a]	390	2.6×10^5
REN-ICAM	YN1	8.7×10^5

[a]From Chacko et al PloS One (ref 31)

Supplemental Figure 4. Quantification of ICAM and PECAM binding sites on transfected REN cells. a. Radioimmunoassay of ^{125}I-labeled anti-ICAM antibody (clone YN1) on REN-ICAM vs. REN wild type cells. b. Comparison of number of 390 (anti-PECAM) binding sites on REN-PECAM cells and YN1 (anti-ICAM) binding sites on REN-ICAM cells.
