# Supplementary material for: Vascular Immunotargeting to Endothelial Determinant ICAM-1 Enables Optimal Partnering of Recombinant scFv-Thrombomodulin Fusion with Endogenous Cofactor
Source: PLoS One. 2013 Nov 14;8(11):e80110. doi: 10.1371/journal.pone.0080110 (PMC3828233; doi:10.1371/journal.pone.0080110)
Supplement: Figure S4 — Quantification of ICAM and PECAM binding sites on transfected REN cells. (PDF) [file pone.0080110.s004.pdf]

Figure S3

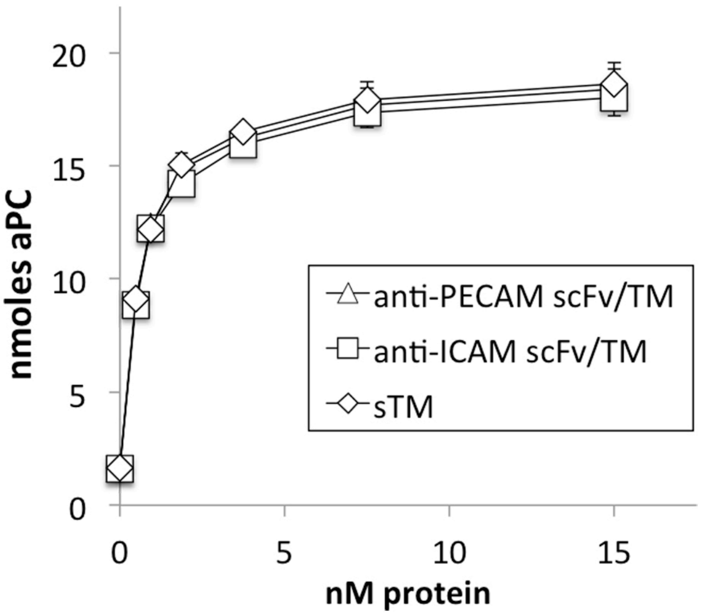

Supplemental Figure 3. Soluble APC generation. To confirm that fusion with anti-PECAM and anti-ICAM scFv's did not adversely affect TM domain function, thrombin-dependent activation of protein C was measured in solution and compared to soluble TM (sTM). The proteins were mixed with 0.5nM thrombin and a large excess of protein C (1μM). The reaction was stopped after 15min by addition of hirudin. The fusion proteins performed identically to sTM over a range of concentrations.
