# Supplementary material for: Vascular Immunotargeting to Endothelial Determinant ICAM-1 Enables Optimal Partnering of Recombinant scFv-Thrombomodulin Fusion with Endogenous Cofactor
Source: PLoS One. 2013 Nov 14;8(11):e80110. doi: 10.1371/journal.pone.0080110 (PMC3828233; doi:10.1371/journal.pone.0080110)
Supplement: Figure S5 — Quantification of EPCR binding sites on transfected REN cells. (PDF) [file pone.0080110.s005.pdf]

Figure S5

a

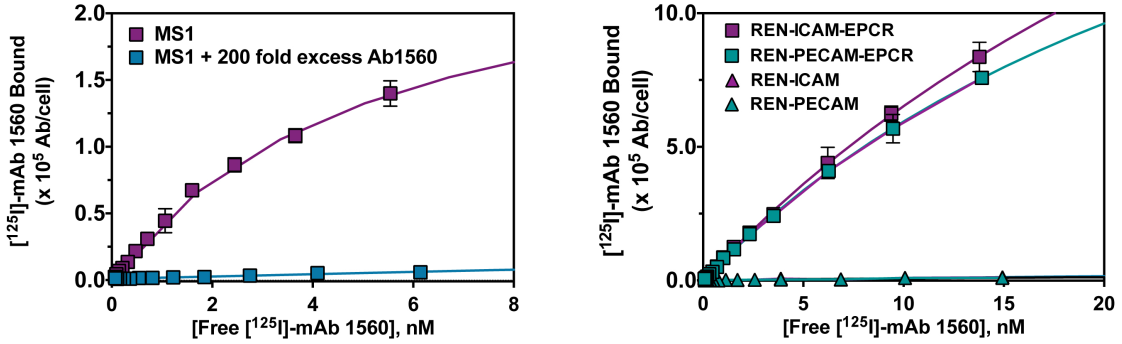

b

Cells	K_d (nM)	B_{max} (Ab/cell)
MS1	5.3	2.7×10^5
REN-ICAM-EPCR	42.2	3.4×10^6
REN-PECAM-EPCR	32.4	2.5×10^6

Supplemental Figure 5. Quantification of EPCR binding sites on transfected REN cells. a. Radioimmunoassay of ^{125}I-labeled anti-EPCR antibody (clone 1560) on MS1, REN-ICAM-EPCR and REN-PECAM-EPCR cells. MS1 cells, which have an order of magnitude less binding sites per cell, are displayed on a separate graph. b. Summary of anti-EPCR binding parameters on each cell type.
