# Supplementary material for: Vascular Immunotargeting to Endothelial Determinant ICAM-1 Enables Optimal Partnering of Recombinant scFv-Thrombomodulin Fusion with Endogenous Cofactor
Source: PLoS One. 2013 Nov 14;8(11):e80110. doi: 10.1371/journal.pone.0080110 (PMC3828233; doi:10.1371/journal.pone.0080110)
Supplement: Figure S6 — APC generation on MS1 cells following blockade of endogenous TM. (PDF) [file pone.0080110.s006.pdf]

Figure S6

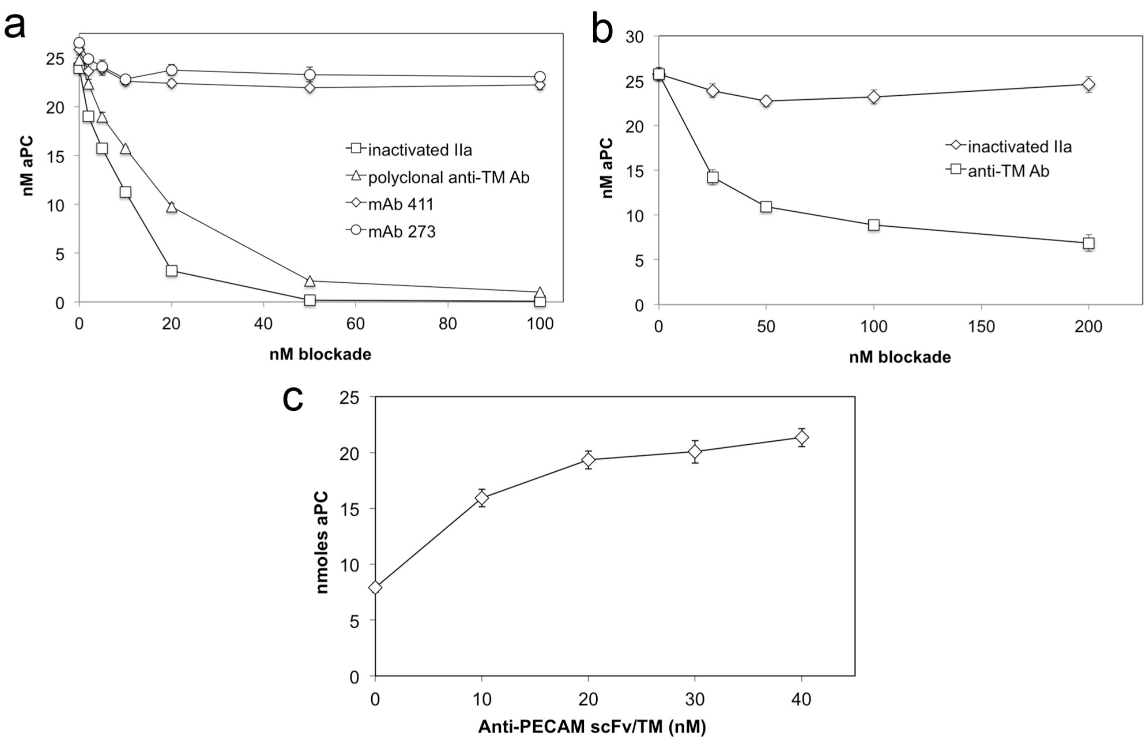

Supplemental Figure 6. APC generation on MS1 cells following blockade of endogenous TM. a. Blockade of endogenous TM with PPACK-inactivated thrombin and polyclonal anti-TM prevents thrombin-dependent APC generation. Monoclonal anti-TM antibodies, clone 411 and clone 273 (generously provided by Dr. Stephen J. Kennel, University of Tennessee, Knoxville, TN) did not inhibit APC generation. Cells were pre-incubated with one of several antibodies or inactivated thrombin x 1 hr prior to addition of thrombin and protein C. b. Antibody blockade, but not inactivated IIa, had a sustained blocking effect when cells were washed prior to addition of thrombin and protein C. c. Antibody blockade of endogenous TM enabled measurement of dose responsive, fusion protein-dependent protein C activation. Cells were treated with 200nM anti-TM antibody and washed, as above, then incubated with anti-PECAM scFv/TM fusion protein x 30 min, prior to addition of thrombin and protein C.
